# Assessing the robustness of passive scattering proton therapy with regard to local recurrence in stage III non-small cell lung cancer: a secondary analysis of a phase II trial

**DOI:** 10.1186/1748-717X-9-108

**Published:** 2014-05-06

**Authors:** Zhengfei Zhu, Wei Liu, Michael Gillin, Daniel R Gomez, Ritsuko Komaki, James D Cox, Radhe Mohan, Joe Y Chang

**Affiliations:** 1 Department of Radiation Oncology, The University of Texas MD Anderson Cancer Center, Houston, TX, USA; 2 Departments of Radiation Physics, The University of Texas MD Anderson Cancer Center, Houston, TX, USA; 3 Current address: Department of Radiation Oncology, Fudan University Shanghai Cancer Center, Shanghai, China

**Keywords:** Non-small cell lung cancer, Proton therapy, Passive scattering proton therapy, Robustness analysis, Worst-case scenario method

## Abstract

**Background:**

We assessed the robustness of passive scattering proton therapy (PSPT) plans for patients in a phase II trial of PSPT for stage III non-small cell lung cancer (NSCLC) by using the worst-case scenario method, and compared the worst-case dose distributions with the appearance of locally recurrent lesions.

**Methods:**

Worst-case dose distributions were generated for each of 9 patients who experienced recurrence after concurrent chemotherapy and PSPT to 74 Gy(RBE) for stage III NSCLC by simulating and incorporating uncertainties associated with set-up, respiration-induced organ motion, and proton range in the planning process. The worst-case CT scans were then fused with the positron emission tomography (PET) scans to locate the recurrence.

**Results:**

Although the volumes enclosed by the prescription isodose lines in the worst-case dose distributions were consistently smaller than enclosed volumes in the nominal plans, the target dose coverage was not significantly affected: only one patient had a recurrence outside the prescription isodose lines in the worst-case plan.

**Conclusions:**

PSPT is a relatively robust technique. Local recurrence was not associated with target underdosage resulting from estimated uncertainties in 8 of 9 cases.

## Introduction

Radiotherapy has an important role in the management of locally advanced non-small cell lung cancer (NSCLC) [1]. However, local control rates after conventional-dose radiotherapy remain disappointing [2] despite concurrent use of chemotherapy. Dose escalation has the potential of improving local control as well as survival if the higher doses can be delivered safely. The physical characteristics of protons allow substantial reductions in the radiation dose to normal tissues while maximizing the dose to the tumor [3,4]. Indeed, we have published promising preliminary results from a prospective phase II study of proton therapy to a prescribed dose of 74 Gy(RBE), at 2 Gy(RBE) per fraction, with concurrent carboplatin-paclitaxel chemotherapy for stage III NSCLC [5].

Although protons can potentially spare more critical structures than photons can, proton therapy is more sensitive to uncertainties because of the tissue-density-dependent finite range of proton beams. The density variations can be induced by all forms of uncertainties, including those associated with set-up, organ motion, and the range uncertainty that is caused by CT number and proton stopping power uncertainties. Plans that do not adequately account for uncertainties (i.e., are insufficiently robust) may be of questionable reliability and lead to unforeseen outcomes such as local failure and increased toxicity to normal tissues. Thus assessing the robustness of proton-therapy treatment plans is an essential part of the plan-evaluation process.

Several strategies have been developed to deal with robustness issues in proton therapy [6-11], among which the worst-case scenario method has been used the most extensively [12]. This method, first introduced by Lomax [6], involves simulating different uncertainty scenarios and incorporating them into the treatment planning process; we have used this method to assess the robustness of intensity-modulated proton therapy (IMPT) plans in clinical practice at our institution [7,8]. However, to the best of our knowledge, this method has not been applied for passive scattering proton therapy (PSPT), nor have potential correlations between plan robustness and local failure been reported. Here, in the first such report, we retrospectively evaluated the robustness of PSPT plans for patients who had experienced local recurrence of NSCLC after PSPT; we used the 4D worst-case robustness quantification method and compared the worst-case isodose lines with the location of the recurrence.

## Methods

### Patients and treatment

Patients in this secondary analysis were a subset of 44 patients with histologically or cytologically proven stage III NSCLC (AJCC 2002) enrolled in a prospective phase II trial of concurrent chemotherapy and proton therapy [5] (clinicaltrials.gov identifier NCT00495170). The protocol has been approved by IRB as a phase II clinical study. Chemotherapy consisted of paclitaxel and carboplatin, given as weekly intravenous infusions of 50 mg/m^2^ paclitaxel and 2-area-under-the-curve units of carboplatin. Proton therapy was delivered as PSPT as described elsewhere [3,5,13] to a total dose of 74 Gy(RBE) in 37 fractions at 2.0 Gy(RBE) per fraction, once per day, and five fractions per week. Clinical characteristics of the patients are summarized elsewhere [5,14].

### 4D Worst-case scenario method and robustness analysis

Plan robustness was analyzed for 9 patients from the phase II trial who experienced local recurrence by using the 4D worst-case scenario method. We have reported a variant of this method that accounts for uncertainties in set-up and range but not organ motion [7,8]. In the current study, we also incorporated the potential influence of intrafractional respiration-related motion into the worst-case scenarios. For each case, the dose distributions were first recalculated at two breathing phases: T0 (end-inspiration) and T50 (end-expiration) on the 4D CT scans used for simulation, using the same PSPT plan as was actually used for treatment (original plan). Then, we computed 9 different dose distributions for each phase: the nominal dose distribution (i.e., that with no consideration of uncertainties) and dose distributions incorporating (a) set-up uncertainties, obtained by shifting the isocenter of the CT images by ±5 mm along the anterior-posterior, left-right, and superior-inferior directions (yielding six dose distributions), and (b) range uncertainty, by scaling the relative stopping power ratios to water by ±3.5% (yielding additional two dose distributions). The uncertainty values for set-up (5 mm) and range (3.5%) were selected based on those used for PSPT planning for lung cancer in our clinical practice [3,5,13]. The worst-case dose distribution in every phase was obtained by assigning the lowest of the 9 doses to each voxel within the clinical target volume (CTV) and the highest of the 9 doses to each voxel outside the CTV. The 4D worst-case dose for the patient was then derived via accumulating the worst-case dose from T0 to T50 phase by using a symmetric force demon registration method [15]. Plans were evaluated by using the same delineations and definitions of structures as were used in the original plan.

To visually evaluate the robustness of the PSPT plans, we extracted the 4D highest (“hot”) and lowest (“cold”) dose values in each voxel from robustness analysis and then plotted “banded” dose-volume histograms (DVHs), which were bounded by the DVHs for the cold and hot doses to CTV. The width of the DVH bands corresponds to the robustness of the plan for the target. We further compared the target coverage of the nominal and 4D worst-case dose distribution by using two common parameters for analyzing target coverage: V_prescription-dose_ (percentage of the volume receiving at least the prescribed dose) and D_95_ (the lowest dose received by 95% of the target). Because we considered and incorporated uncertainties in the worst-case plans, using a PTV margin (typically introduced to account for uncertainties) would not be appropriate. Therefore, we chose to evaluate the PTV as the target volume for the nominal plan but the CTV as the target volume for the worst-case plan.

### Correlations between local recurrence and the worst-case dose distribution

For each patient, the 4D worst-case dose distribution was displayed on the average simulation CT, with the isodose lines from 60 to 74 Gy(RBE) at a step size of 2 Gy(RBE). For the purposes of this study, local recurrence was defined as disease relapse within the PTV on the average simulation CT at any time, regardless of the timing of previous failures. The location and volume of the recurrent lesion relative to the 4D worst-case dose distribution were evaluated on PET scans on which the recurrences were first identified, fused with the average simulation CT scan.

## Results

### Update of the clinical outcomes

**Figure 1:**
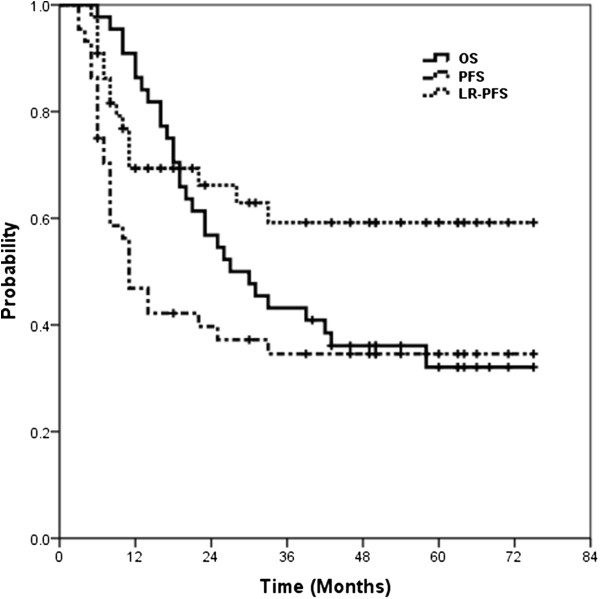
Kaplan–Meier curves for overall survival (OS), progression-free survival (PFS), and locoregional-progression-free survival (LR-PFS).

Median follow-up time for all patients in the trial was 28.5 months (range 6-75 months). Median survival time was 30 months (95% confidence interval [CI] 16-38); overall survival (OS) rates were 43.2% at 3 years and 32.1% at 5 years. The median time to progression was 11 months (95% CI 7-15), with progression-free survival [PFS] rates of 34.6% at both 3 years and 5 years. Locoregional recurrence-PFS (LR-PFS) rates were 59.2% at both 3 years and 5 years (Figure 1).

When this analysis was undertaken, a total of 28 patients had experienced relapse after treatment, 21 with distant metastasis and 16 with locoregional recurrence as a component of failure. Among the 16 patients with locoregional recurrence, recurrence was local only in 4, regional only in 3, both local and regional in 7, and unknown for 2 (no images were available from outside hospitals).

### Robustness analysis

Robustness analyses were undertaken for 9 of the 11 patients with local recurrence, because the original treatment plans for the other 2 patients had been lost during an update of the treatment planning system. One of these 9 patients received adaptive planning and treatment [14] owing to significant shrinkage of the tumor over the course of treatment. The prescription doses for the first and second (adaptive) plans for that patient were 44 Gy and 30 Gy, and we generated two 4D worst-case dose distributions and evaluated them separately.

**Figure 2:**
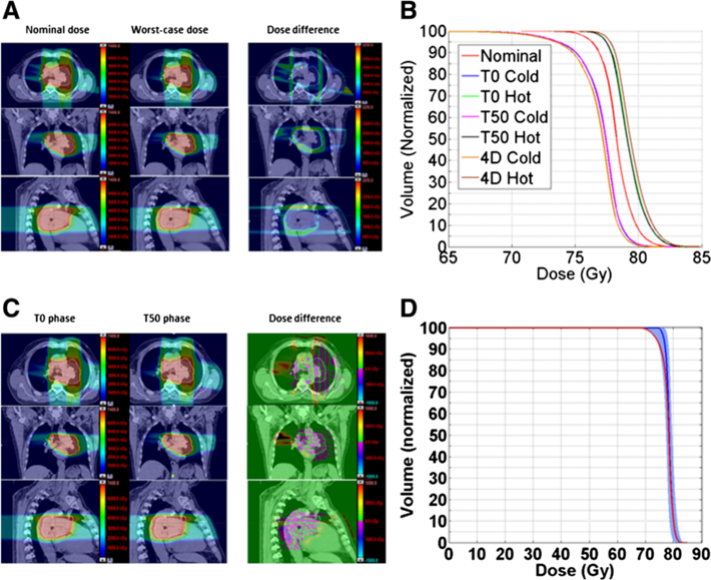
**Robustness analyses for a representative case. (A)** Axial, sagittal, and coronal views of the dose distributions for the nominal and worst-case plans and the difference in dose between them (nominal minus worst-case). The white line represents the contoured clinical target volume (CTV). **(B)** Dose-volume histogram of the CTV for seven scenarios: the nominal plan and the cold and hot doses on the T0 phase, T50 phase, and 4D accumulated plans. **(C)** Worst-case dose distributions on the T0 and T50 phases and the differences between them. **(D)** Banded dose-volume histogram of the CTV. The blue and red solid lines represent the DVHs for the CTV (blue) and the PTV (red) in the nominal plan.

The dose distributions and DVHs used in robustness analysis of a representative case are shown in Figure 2. The volume enclosed by the prescription isodose line in the 4D worst-case dose distribution was smaller than that in the nominal dose distribution, but inside the target volume no significant dose difference was found (Figure 2A), indicating that the uncertainties perturbed the doses mostly around the marginal regions around target for PSPT. Figure 2B shows that the DVHs of the hot and cold doses on the T0 and T50 phases overlap almost completely, suggesting that the impact of set-up and range uncertainty on CTV coverage is almost the same between different respiratory phases. However, a slight shift was noted between the 4D accumulated cold and hot doses and those of the T0/T50 phases resulting from the dose accumulation that followed the organ motion from the T0 to T50 phases. The dose distributions were different both inside and outside CTV between the T0 and T50 phases (Figure 2C), but the extent of that difference was generally not significant, especially within the CTV. The banded DVHs of CTV is shown on Figure 2D, the narrowness of the band implies that the PSPT plan was not particularly sensitive to the treatment uncertainties.

For every patient, the target coverage of the 4D worst-case dose distribution was slightly lower than that in the nominal dose distribution, with median differences of 2.8% (range, 0.5%-4.4%) for V_prescription-dose_ and 1.9 Gy (range, 0-3.5 Gy) for D_95_ (Table 1). The comparison of the DVHs for the CTV in the 4D worst-case dose distribution (the lowest edge of the blue shadow) and for the PTV in the nominal dose distribution (red line) for the selected case is also shown in Figure 2D.

**Table 1:** Target coverage in the nominal and worst-case plans

	**V**_**prescription dose **_**(%)**			**D**_**95 **_**(Gy)**		
	**To PTV (nominal plan)**	**To CTV (worst-case)**	**Difference**	**To PTV (nominal plan)**	**To CTV (worst-case)**	**Difference**
Patient 1	90.7	89.1	1.6	72.0	70.9	1.1
Patient 2	93.9	93.4	0.5	73.4	73.4	0
Patient 3	95.6	91.1	3.5	74.2	72.7	1.5
Patient 4	90.0	85.6	4.4	69.2	66.9	2.3
Patient 5	81.0	78.0	3.0	68.9	65.4	3.5
Patient 6	88.1	83.0	4.1	67.3	64.8	2.5
Patient 7	93.0	91.0	2.0	72.0	70.8	1.2
Patient 8	81.1	78.5	2.6	69.4	67	2.4
Patient 9^§^						
1^st^ plan	95.1	93.1	2.0	44.0	43.6	0.4
2^nd^ plan	97.6	93.6	4.0	30.3	29.8	0.5

*Abbreviations:V*_*prescription dose*_ volume (in percent) receiving at least the prescription dose, *D*_*95*_ the minimum dose received by 95% of the target, *PTV* planning target volume, *CTV* clinical target volume.

^§^Two plans were prepared for this patient during the radiation therapy (“adaptive planning”); the prescribed doses were 44 Gy in the first plans and 30 Gy in the second.

**Figure 3:**
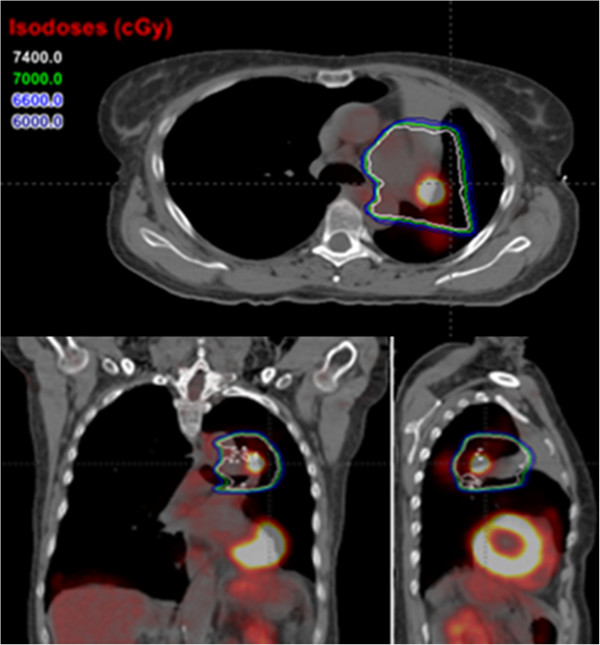
Axial, coronal, and sagittal views of fused PET/CT scans showing a local recurrence that occurred within both the 74 Gy(RBE) (white) isodose lines on the worst-case dose distribution.

**Figure 4:**
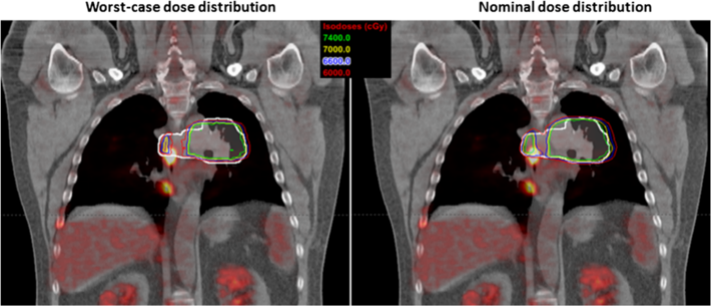
Worst-case (left) and nominal (right) dose-distribution plans for the patient whose local recurrence appeared outside the 60 Gy(RBE) (red) isodose lines on both set of plans.

The local recurrences appeared within the volume enclosed by the prescription-dose line of the worst-case dose distribution in 8 of the 9 patients (c.f. Figure 3), including the patient who received the adaptive treatment (the lesion was covered in both the original and adaptive plans). The 9th patient had a local recurrence in a subcarinal lymph node, outside the 60 Gy(RBE) isodose lines on the worst-case dose distribution (Figure 4). However, that node was also outside the 60 Gy(RBE) isodose line in the nominal dose distribution (Figure 4).

## Discussion

In this study, we confirmed that dose distributions could be perturbed by set-up, organ-motion, and range uncertainties in proton therapy, and we further found that any underdosed regions of the CTV in the 4D worst-case dose distribution of PSPT always occurred around the edges of the CTV. This finding was consistent with the characteristics of PSPT, in which the per-field dose is delivered by summing the Bragg peaks from different mono-energetic protons by using range modulation wheels or primary and secondary scatters to produce a uniform dose distribution to cover the whole target per field (the spread-out Bragg peak [SOBP]). The water-equivalent length of the plateau part of the SOBP of this field is determined by the target size penetrated by this field. Compensators are used for each individual field to achieve distal dose conformity in a target volume. Apertures are also used for each individual field to laterally shape the dose distribution to protect the critical normal tissues nearby. The total dose is then formed by fields from different beam angles. The distal and proximal edges of the SOBPs are determined based on the shapes of target volumes and the compensator design, so changes of the densities along the beam pathways can shift the position of edges, possibly resulting in underdoses at the marginal regions of targets. However, the doses to the middle of targets remain unchanged because the magnitude of the SOBP is not disturbed.

In this study, we compared the target dose coverage between the nominal and worst-case dose distributions, reasoning that it could be a good indicator of the plan’s robustness because the introduced uncertainties would lead to target underdosage. Some treatment planning techniques have already incorporated ways of accounting for the set-up and range uncertainties in PSPT; for example, patient set-up uncertainties are addressed by expanding the aperture, and range uncertainty by smearing the compensator and by using appropriate beam-specific distal and proximal margins. Our discovery in this study that the target dose coverage of the worst-case dose distribution was smaller than the nominal dose distribution confirmed the negative effect of uncertainties on the dose distribution. However, we also observed that this negative effect was small, meaning that the influence of the uncertainties on the dose distribution in PSPT was not significant. Moreover, the analysis of banded DVHs for the CTV (Figure 2D) also suggested that the PSPT plan was not sensitive to uncertainties [16]. Collectively, these results demonstrate that our methods can effectively account for uncertainties in patient set-up and proton range, and thus that PSPT can be considered relatively robust with regard to these uncertainties.

In our clinical practice for PSPT planning, the change in tissue density due to breathing motion is mitigated by use of averaged 4D CT and integrated-GTV (GTV over all phases) density override (i.e., assignment of maximum CT HU number from individual phases). This method is considered state-of-the-art for accounting for intrafractional motion, and we have been shown it to be effective for mitigating the influence of breathing motion in PSPT [17]. In addition, a specified planning process is also used to deal with respiration-induced motion for PSPT. In this process, we first generate the original plan on the average simulation CT and then create two “verification” dose distributions by recalculating the dose on the 4D simulation CT scans at two extreme breathing phases (T0 and T50) using the original plan. The original plan is then adjusted until the verification and original dose distributions all meet the required prescription criteria. Our finding of little difference in target coverage in the worst-case dose distributions between the two phases (Figure 2B) could reflect the effectiveness of the approaches we applied to address respiration-induced tumor motion. However, the fact that there is a difference in the 4D accumulated worst-case doses from the T0/T50 phases suggests that respiratory motion should be included in robustness analyses.

For 8 of the 9 patients in this study, the volumes of the recurrent lesions were covered by the prescription dose on the worst-case dose distribution, suggesting that these recurrences were unlikely to have been caused by the uncertainties-induced underdosing; this finding also indicates that the approaches we used for dealing with the uncertainties were quite satisfactory for PSPT. One patient did experience recurrence beyond the region enclosed by the 60 Gy(RBE) isodose line in the worst-case dose distribution; however, this lesion was also outside the region enclosed by the 60 Gy(RBE) isodose line on the nominal dose distribution (Figure 4), suggesting this relapse was not simply related to the dose missing resulting from uncertainties. Because the primary lesion in this patient abutted the esophagus, target coverage was compromised during treatment planning because of concerns about esophageal toxicity. This example emphasizes the importance of target dose coverage for local control, and it also suggests that a complicated delivery technique such as IMPT which might be able to spare the normal tissue better would be better suited for complex cases such as this one [4].

Finally, the incidence of local recurrence (25%) in the phase II trial of PSPT for lung cancer on which this analysis was based indicates that the prescribed dose of 74 Gy(RBE) may not be enough to eliminate some inherently radiation-resistant NSCLC tumor cells. A growing body of evidence [18] now suggests that tumor recurrence is associated with failure to eradicate cancer stem cells, which are tumorigenic, capable of self-renewal, and relatively radioresistant. Strategies that can enhance the biological effects of radiation include further dose escalation, use of new dose delivery techniques (e.g., IMPT), modification of dose-fractionation schedules (e.g., hypofractionated radiotherapy [19] or integrated boost techniques), and use of radiation-sensitizing agents such as molecular targeted therapy. The effect of these approaches on cancer stem cells needs further investigation.

## Conclusions

The dose distribution of PSPT plan was affected by the treatment uncertainties, with the underdose area mainly located on the target margin. The target dose coverage of the worst-case dose distribution was slightly smaller than the nominal dose distribution, indicating the negative effect of uncertainties on the dose distribution which was not significant in PSPT. Moreover, the analysis of banded DVHs for the CTV suggested that the PSPT plan was not sensitive to uncertainties. Thus, PSPT is a relatively robust technique. Local recurrence was not associated with target missing due to the robustness issue of PSPT in most cases in our study.

## Abbreviations

CI: Confidence interval; CTV: Clinical target volume; D95: The lowest dose received by 95% of the target; DVHs: Dose-volume histograms; IMPT: Intensity-modulated proton therapy; LR-PFS: Locoregional recurrence-PFS; NSCLC: Non-small cell lung cancer; OS: Overall survival; PET: Positron emission tomography; PFS: Progression-free survival; PSPT: Passive scattering proton therapy; PTV: Planning target volume; SOBP: Spread-out Bragg peak; Vprescription-dose: Percentage of the volume receiving at least the prescribed dose.

## Competing interests

The authors declare that they have no competing interests.

## Authors’ contributions

ZZ, WL and JYC carried out the design of the study, participated in data analysis and manuscript writing. ZZ also contributed to study coordinator. WL also contributed to generate the worst-case plan and participate in acquisition of data. MG, DRG, RK, JDC and RM helped study design and revised the manuscript. All authors read and approved the final manuscript.
